# Nirmatrelvir/ritonavir reduced mortality in severe or critical COVID-19 patients: a multicenter retrospective cohort study

**DOI:** 10.3389/fmed.2026.1747565

**Published:** 2026-02-20

**Authors:** Heng Zhao, Wanting Meng, Xing Lv, Shaokui Si, Shunjun Wu, Jing Li, Zhigui Cai, Ruoqi Jin, Ling Yang, Chenyao Li, Liqiang Song

**Affiliations:** 1 Department of Pulmonary and Critical Care Medicine, Xijing Hospital of Air Force Medical University, Xi’an, China; 2 Department of Pulmonary and Critical Care Medicine, The 987th Hospital of the PLA Joint Service Support Force, Baoji, China

**Keywords:** COVID-19, critical illness, mortality, nirmatrelvir/ritonavir, severe disease

## Abstract

**Background:**

Patients with severe or critical coronavirus disease 2019 (COVID-19) remain at a high risk of mortality. Although nirmatrelvir/ritonavir has demonstrated efficacy in non-severe COVID-19 patients with high-risk factors, its effectiveness in hospitalized patients with severe or critical COVID-19 remains unclear. This study evaluates the effectiveness of nirmatrelvir/ritonavir in this specific population.

**Methods:**

In this multicenter retrospective cohort study, we included adults hospitalized with severe or critical COVID-19 at three tertiary hospitals in Shaanxi Province between December 2022 and November 2023. Participants were non-randomly categorized into either the nirmatrelvir/ritonavir group or the non-antiviral group based on whether they received nirmatrelvir/ritonavir during hospitalization. The primary outcome was 28-day mortality, and secondary outcomes included in-hospital mortality and post-baseline hospitalization duration.

**Results:**

Among the 386 patients (nirmatrelvir/ritonavir group, *n* = 173, and non-antiviral group, *n* = 213), those in the nirmatrelvir/ritonavir group had significantly lower 28-day mortality than those in the non-antiviral group (6.9% vs. 15.5%; *p* = 0.002). Additionally, the nirmatrelvir/ritonavir group had reduced in-hospital mortality rates (8.1% vs. 16.0%; *p* = 0.003). After multivariable adjustment, the use of nirmatrelvir/ritonavir remained independently associated with a reduced risk of 28-day mortality (adjusted hazard ratio (aHR) = 0.346, 95% confidence interval (CI): 0.175–0.687; *p* = 0.002) and in-hospital mortality (aHR = 0.374, 95% CI: 0.196–0.716; *p* = 0.003). Subgroup analyses suggested that the reduced mortality risk was particularly evident in patients aged ≥65 years, non-smokers, those without chronic lung disease or hypertension, those with critical illness, and those who initiated treatment within 5 days of symptom onset. The median post-baseline hospitalization duration was longer in the nirmatrelvir/ritonavir group than in the non-antiviral group (11.0 vs. 9.0 days; *p* = 0.019).

**Conclusion:**

Nirmatrelvir/ritonavir was associated with significantly reduced mortality in patients hospitalized with severe or critical COVID-19, supporting its clinical use in this population.

## 1 Introduction

Coronavirus disease 2019 (COVID-19) continues to pose a significant global health threat and remains associated with a substantial disease burden. According to World Health Organization data, there were approximately 3.51 million newly reported cases and over 67,000 deaths worldwide in 2024 (1). The clinical spectrum of COVID-19 ranges from acute infection to post-COVID-19 conditions, making its management an important clinical challenge (2–4). During the acute phase of infection, some patients required hospitalization due to COVID-19, and among those hospitalized, a substantial proportion progressed to severe or critical illness and faced a high risk of mortality. During the Omicron-dominant phase, an estimated 18.5 to 32% of hospitalized COVID-19 patients progressed to severe or critical illness (5, 6). Importantly, mortality rates among these severe or critical patients continued to exceed 30% in the majority of reports (7–11). Therefore, developing and implementing effective strategies to reduce mortality among severe or critical COVID-19 patients remains a vital clinical and public health priority.

The viral load of severe acute respiratory syndrome coronavirus 2 (SARS-CoV-2) is strongly correlated with disease severity and mortality, with higher viral loads generally associated with worse outcomes (12–17). As a result, effective suppression of viral replication is a critical component of COVID-19 treatment. Nirmatrelvir/ritonavir (also known as Paxlovid) is an oral co-packaged antiviral agent. Nirmatrelvir can inhibit the SARS-CoV-2 main protease, while ritonavir acts as a pharmacokinetic enhancer by inhibiting cytochrome P450 3A (CYP3A). This action slows down the metabolism of nirmatrelvir and extends its therapeutic plasma levels (18). The EPIC-HR trial demonstrated that, for non-hospitalized high-risk COVID-19 patients, initiating nirmatrelvir/ritonavir treatment within 3 or 5 days of symptom onset reduced the composite risk of COVID-19-related hospitalization or death within 28 days by 88.9 and 87.8%, respectively, compared to placebo, and also accelerated viral clearance (19). Subsequent studies have further confirmed its sustained efficacy in non-severe patients infected with the Omicron variant (20–22).

However, the effectiveness of nirmatrelvir/ritonavir in patients with severe or critical COVID-19 remains uncertain, and the available evidence is limited and contradictory. For instance, Yang et al. reported a reduction in 28-day mortality among critical COVID-19 patients receiving invasive mechanical ventilation who were treated with nirmatrelvir/ritonavir (23), and Chen et al. observed improved 28-day survival in severe COVID-19 patients aged 80 years or older who received the treatment (24); however, two other studies found no significant survival benefit associated with the use of nirmatrelvir/ritonavir in severe or critical COVID-19 patients (25, 26). To address this ongoing controversy and provide more robust evidence, we conducted a multicenter retrospective cohort study at three tertiary hospitals in Shaanxi Province to evaluate the efficacy of nirmatrelvir/ritonavir in hospitalized adults with severe or critical COVID-19.

## 2 Patients and methods

### 2.1 Study design and ethical approval

This was a multicenter retrospective cohort study. The study protocol was reviewed and approved by the Medical Ethics Committee of the First Affiliated Hospital of Air Force Medical University (Xijing Hospital) (Approval No.: KY20222118-F-1), and the requirement for informed consent was waived due to the retrospective nature of the study. The study was conducted in accordance with the STROBE guidelines.

### 2.2 Study population

Adult patients hospitalized with severe or critical COVID-19 at three tertiary general hospitals in Shaanxi Province between 1 December 2022 and 30 November 2023 were eligible for inclusion. The diagnostic criteria for severe or critical COVID-19 were based on the Diagnostic and Treatment Protocol for Novel Coronavirus Infection (Trial Version 10) issued by the National Health Commission of China (27). Severe COVID-19 was defined as meeting any of the following criteria: Respiratory rate of ≥30 breaths/min, oxygen saturation level of ≤93% at rest, or arterial partial pressure of oxygen to fraction of inspired oxygen ratio of ≤300 mmHg. Critical COVID-19 was defined as respiratory failure that necessitates mechanical ventilation, shock, or other organ failure requiring intensive care. Complete definitions are provided in Supplementary Table S1.

The inclusion criteria were as follows: (i) laboratory confirmation of SARS-CoV-2 infection by a positive nucleic acid or antigen test and (ii) fulfillment of the diagnostic criteria for severe or critical COVID-19. The exclusion criteria were as follows: (i) age <18 years, (ii) receipt of any antiviral drugs (including nirmatrelvir/ritonavir, azvudine, or molnupiravir) before the baseline date, and (iii) use of other antiviral drugs (including azvudine and molnupiravir) during the observation period after baseline.

### 2.3 Data collection

Clinical data were retrospectively extracted from the electronic medical record systems of the three participating hospitals by three independent data collectors. To improve accuracy and consistency, all extracted data were reviewed and verified by two additional reviewers. The following variables were collected: (i) demographics, including sex, age, and smoking history; (ii) prior SARS-CoV-2 infection; (iii) comorbidities, including chronic lung disease, hypertension, heart disease, cerebrovascular disease, diabetes, chronic liver disease, chronic kidney disease, cancer, and immunocompromised status (immunodeficiency disorders or long-term use of glucocorticoids/immunosuppressants); (iv) disease course, including the date of COVID-19 symptom onset and the date of hospital admission; (v) treatments received before admission; (vi) disease severity, including changes in COVID-19 severity during hospitalization and the corresponding dates; (vii) inpatient antiviral therapy, including the name, dosage, and start and end dates of antiviral drugs administered during hospitalization; (viii) concomitant therapies during hospitalization, including glucocorticoids, antibiotics, tocilizumab, baricitinib, and anticoagulants; (ix) SARS-CoV-2 nucleic acid test results; and (x) clinical outcomes, including the date of discharge and the discharge status (improved, deceased, or other).

### 2.4 Exposure

The exposure of interest was treatment with nirmatrelvir/ritonavir during the hospitalization period following a diagnosis of severe or critical COVID-19. Based on this exposure, patients were categorized into the nirmatrelvir/ritonavir group and the non-antiviral group.

### 2.5 Baseline date

The baseline date was defined separately for each group. In the nirmatrelvir/ritonavir group, the baseline date was the date of the first dose administered after patients met the criteria for severe or critical COVID-19 during hospitalization (the median time from meeting the criteria to receiving the first dose was 1.0 days, with an interquartile range [IQR] of 0.0–1.0 days). In the non-antiviral group, the baseline date was defined as the date when patients first met the criteria for severe or critical COVID-19 during hospitalization. The end of the observation period was defined as the earliest of hospital discharge, death, or 28 days after the baseline date.

### 2.6 Outcomes

The primary outcome was 28-day mortality, which refers to death from any cause within 28 days from the baseline date while the patient was still hospitalized. Secondary outcomes included in-hospital mortality, defined as death from any cause during hospitalization regardless of its timing in relation to the baseline date, and the length of hospital stay after the baseline date.

### 2.7 Sample size estimation

Epidemiological data indicate that the mortality rate of patients with severe or critical COVID-19 is approximately 30% (11). According to a study by Yang et al. (23), the 28-day mortality rates were 41.9% in the nirmatrelvir/ritonavir group and 77.6% in the control group, resulting in a mortality rate ratio of 0.54. Based on these estimates, we performed a sample size calculation with *β* set at 0.2 (power = 0.8) and a two-sided *α* level of 0.05. The required sample size was estimated to be 143 patients per group, resulting in a total of 286 participants. As the data were obtained from hospitalized patients, no loss to follow-up was observed.

### 2.8 Statistical analysis

Continuous variables were assessed for normality using the Shapiro–Wilk test. As all continuous variables were non-normally distributed, they are presented as medians (IQRs), and between-group comparisons were performed using the Mann–Whitney U test. Categorical variables are presented as numbers (percentages) and were compared using the chi-squared test or Fisher’s exact test, as appropriate. Survival probabilities were visualized using Kaplan–Meier curves, and differences between the groups were evaluated using the log-rank test. Hazard ratios (HRs) and adjusted hazard ratios (aHRs), with 95% confidence intervals (CIs), were estimated using Cox proportional hazards models. To control for potential confounders, multivariable Cox regression models were constructed by including covariates that showed a significant association (*p* < 0.05) in univariate analyses. Candidate covariates included age, sex, smoking history, prior SARS-CoV-2 infection, comorbidities, baseline COVID-19 severity, onset-to-baseline time (defined as the interval from symptom onset to baseline), and concomitant treatments (glucocorticoids, antibiotics, tocilizumab, baricitinib, and anticoagulants) (28). Subgroup analyses were conducted for 28-day mortality and in-hospital mortality according to age (<65 or ≥65 years), sex, presence of chronic lung disease, hypertension, heart disease, diabetes, baseline COVID-19 severity (severe or critical), and onset-to-baseline time (≤5 or >5 days). Interaction effects within the subgroups were formally tested. In addition, to further explain the finding of prolonged hospitalization, we performed a competing risks analysis by estimating cumulative incidence functions for death and discharge, with between-group comparisons conducted using Gray’s test. We also conducted a severity-stratified analysis of hospitalization duration, comparing the groups using the Mann–Whitney U test within the severe and critical subgroups. All statistical analyses were performed using SPSS version 27.0, and figures were generated using R version 4.3.2. A two-sided *p*-value of <0.05 was considered statistically significant.

## 3 Results

### 3.1 Patient characteristics

**Figure 1:**
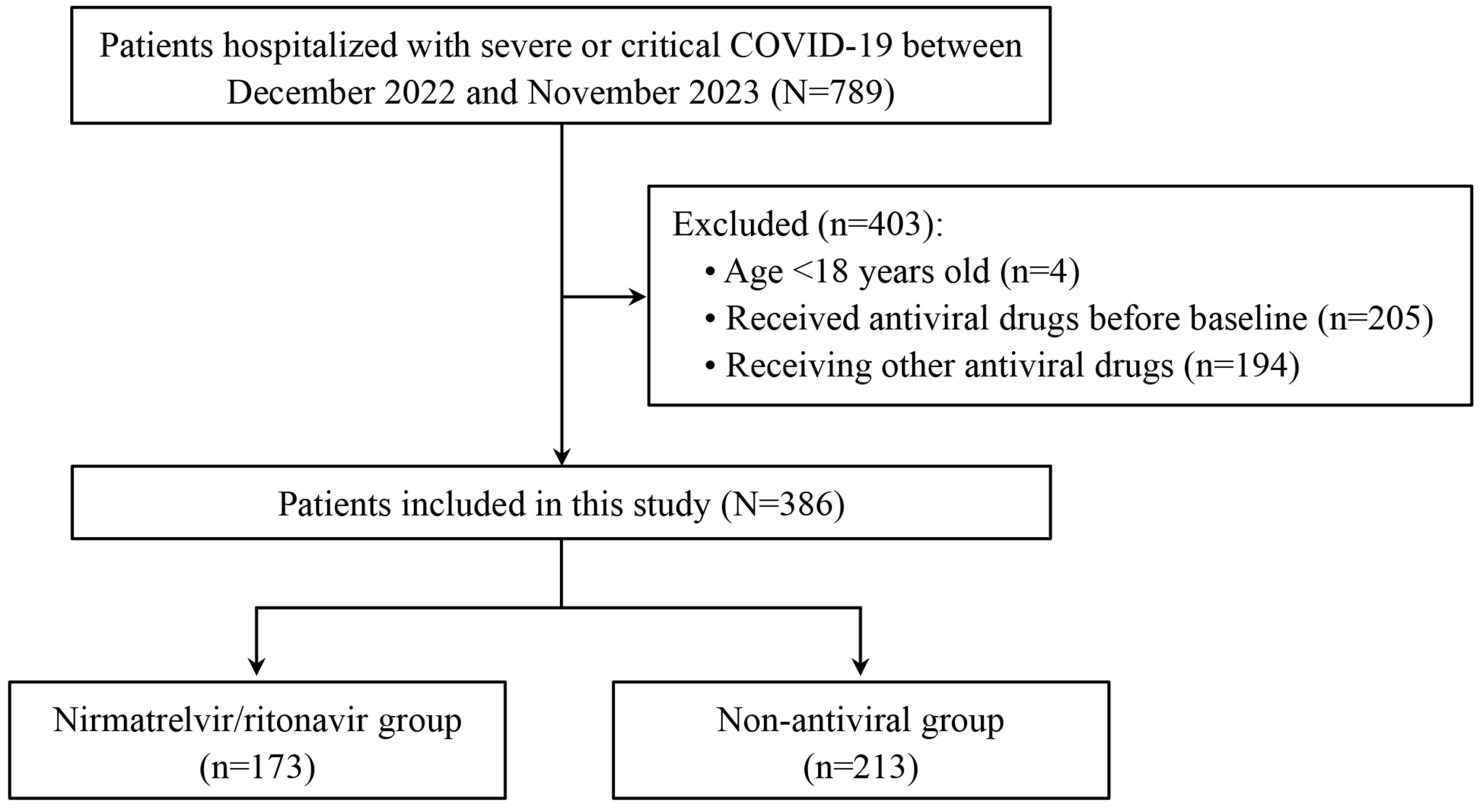
Flowchart of the study.

A total of 789 patients with severe or critical COVID-19 were screened for eligibility. Of these, 403 were excluded, including 4 patients aged <18 years, 205 who received antiviral therapy before baseline, and 194 who received other antiviral agents (azvudine or molnupiravir) after baseline. Ultimately, 386 patients were included in the analysis, with 173 in the nirmatrelvir/ritonavir group and 213 in the non-antiviral group (Figure 1). Baseline characteristics are shown in Table 1. Overall, the cohort had a median age of 73.0 years (IQR: 61.0–83.0 years) and consisted predominantly of men (69.7%). Comorbidities were common (94.0%), with heart disease (64.2%), hypertension (56.0%), and diabetes (36.3%) being the most frequent. At baseline, 129 patients (33.4%) had critical COVID-19. The two groups were generally well balanced across measured baseline characteristics, including demographics, prior SARS-CoV-2 infection, comorbidities, baseline COVID-19 severity, onset-to-baseline time, and concomitant treatments (Table 1).

**Table 1:** Baseline characteristics of patients with severe or critical COVID-19 in the nirmatrelvir/ritonavir group and the non-antiviral group.

Characteristics	Total (*N* = 386)	Nirmatrelvir/ritonavir (*n* = 173)	Non-antiviral (*n* = 213)	*p*-value
Age (years), M (IQR)	73.0 (61.0–83.0)	74.0 (64.5–85.0)	71.0 (59.5–82.0)	0.089
Sex, *n* (%)				0.568
Male individuals	269 (69.7)	118 (68.2)	151 (70.9)	
Female individuals	117 (30.3)	55 (31.8)	62 (29.1)	
Smoking history, *n* (%)	89 (23.1)	38 (22.0)	51 (23.9)	0.646
Prior SARS-CoV-2 infection, *n* (%)	13 (3.4)	5 (2.9)	8 (3.8)	0.639
Comorbidities, *n* (%)	363 (94.0)	160 (92.5)	203 (95.3)	0.245
Chronic lung disease	71 (18.4)	34 (19.7)	37 (17.4)	0.565
Hypertension	216 (56.0)	97 (56.1)	119 (55.9)	0.968
Heart disease	248 (64.2)	106 (61.3)	142 (66.7)	0.271
Cerebrovascular disease	108 (28.0)	52 (30.1)	56 (26.3)	0.412
Diabetes	140 (36.3)	69 (39.9)	71 (33.3)	0.183
Chronic liver disease	51 (13.2)	21 (12.1)	30 (14.1)	0.575
Chronic kidney disease	70 (18.1)	25 (14.5)	45 (21.1)	0.090
Cancer	82 (21.2)	43 (24.9)	39 (18.3)	0.118
Immunocompromised status	21 (5.4)	9 (5.2)	12 (5.6)	0.853
Baseline COVID-19 severity, *n* (%)				0.090
Severe	257 (66.6)	123 (71.1)	134 (62.9)	
Critical	129 (33.4)	50 (28.9)	79 (37.1)	
Onset-to-baseline time (days), M (IQR)	7.0 (2.0–12.0)	8.0 (3.0–12.0)	7.0 (1.0–13.0)	0.106
Concomitant treatments, *n* (%)				
Systemic glucocorticoids	279 (72.3)	133 (76.9)	146 (68.5)	0.069
Antibiotics	309 (80.1)	141 (81.5)	168 (78.9)	0.520
Tocilizumab	9 (2.3)	1 (0.6)	8 (3.8)	0.086
Baricitinib	46 (11.9)	23 (13.3)	23 (10.8)	0.451
Anticoagulants	282 (73.1)	127 (73.4)	155 (72.8)	0.888

M, median; IQR, interquartile range; SARS-CoV-2, severe acute respiratory syndrome coronavirus 2; COVID-19, coronavirus disease 2019.

### 3.2 28-day mortality

**Figure 2:**
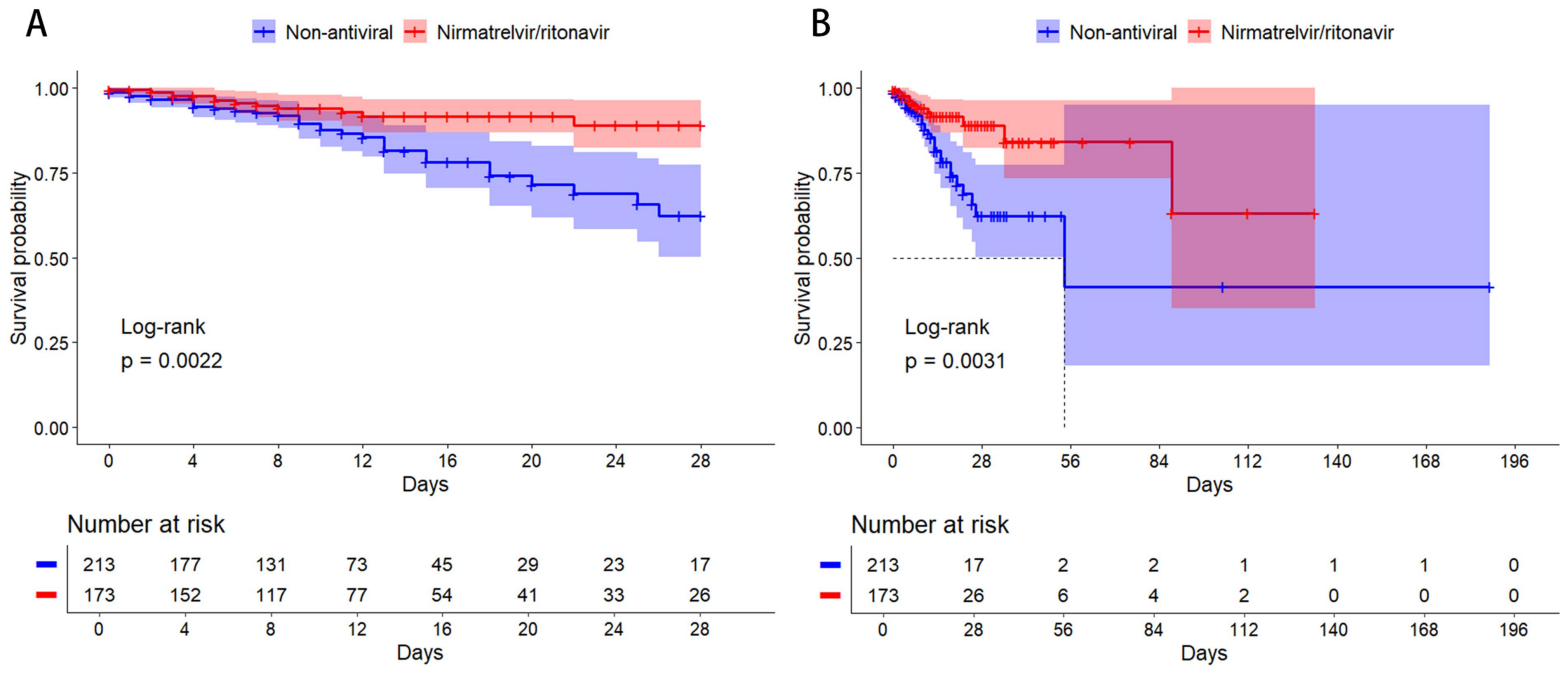
Kaplan–Meier curves for mortality in severe or critical COVID-19: nirmatrelvir/ritonavir vs. non-antiviral. **(A)** 28-day mortality. **(B)** In-hospital mortality. The log-rank test was used to compare the survival distributions between the two groups. The number of patients at risk over time is shown below each graph.

Within 28 days, 45 patients (11.7%) died. The 28-day mortality rate was significantly lower in the nirmatrelvir/ritonavir group than in the non-antiviral group (6.9% vs. 15.5%; *p* = 0.002; Table 2). The Kaplan–Meier curve for 28-day mortality is shown in Figure 2A.

**Table 2:** Clinical outcomes and hospital stay of patients with severe or critical COVID-19 in the nirmatrelvir/ritonavir group and the non-antiviral group.

Outcomes	Total (*N* = 386)	Nirmatrelvir/ritonavir (*n* = 173)	Non-antiviral (*n* = 213)	*p*-value
28-day mortality, *n* (%)	45 (11.7)	12 (6.9)	33 (15.5)	0.002^a^
In-hospital mortality, n (%)	48 (12.4)	14 (8.1)	34 (16.0)	0.003^a^
Post-baseline hospitalization duration (days), M (IQR)	10.0 (6.0–16.0)	11.0 (6.0–19.0)	9.0 (5.0–14.0)	0.019

^a^Log-rank test. M, median; IQR, interquartile range.

Cox regression analyses evaluating the association between nirmatrelvir/ritonavir and clinical outcomes are summarized in Table 3. In univariate analysis, nirmatrelvir/ritonavir treatment was associated with a significantly lower risk of 28-day mortality compared to no antiviral therapy (HR = 0.372, 95% CI: 0.192–0.721; *p* = 0.003), and the full univariate results are provided in Supplementary Table S2. After adjustment for age, heart disease, diabetes, baseline COVID-19 severity, and concomitant tocilizumab use, multivariable Cox regression confirmed that nirmatrelvir/ritonavir remained associated with a significantly reduced risk of 28-day mortality (aHR = 0.346, 95% CI: 0.175–0.687; *p* = 0.002).

**Table 3:** Cox regression analysis of nirmatrelvir/ritonavir and clinical outcomes.

Outcomes	Univariate Cox Regression		Multivariable Cox Regression	
	HR (95%CI)	*p*-value	aHR (95% CI)	*p*-value
28-day mortality				
Non-antiviral	1 (reference)	–	1 (reference)	–
Nirmatrelvir/ritonavir	0.372 (0.192–0.721)	0.003	0.346 (0.175–0.687)	0.002
In-hospital mortality				
Non-antiviral	1 (reference)	–	1 (reference)	–
Nirmatrelvir/ritonavir	0.402 (0.215–0.751)	0.004	0.374 (0.196–0.716)	0.003

Multivariable Cox regression: Adjusted for age, heart disease, diabetes, baseline COVID-19 severity, and concomitant tocilizumab use. HR, hazard ratio; aHR, adjusted hazard ratio; CI, confidence interval.

### 3.3 In-hospital mortality

As shown in Table 2, 48 patients (12.4%) died during hospitalization. In-hospital mortality was significantly lower in the nirmatrelvir/ritonavir group compared to the non-antiviral group (8.1% vs. 16.0%; *p* = 0.003). The corresponding Kaplan–Meier curve is presented in Figure 2B.

In univariate Cox regression, nirmatrelvir/ritonavir was associated with a significantly lower risk of in-hospital mortality (HR = 0.402, 95% CI: 0.215–0.751; *p* = 0.004), with the full results shown in Supplementary Table S3. After adjusting for age, heart disease, diabetes, baseline COVID-19 severity, and concomitant tocilizumab use, multivariable analysis remained consistent and showed a significant reduction in mortality risk associated with nirmatrelvir/ritonavir (aHR = 0.374, 95% CI: 0.196–0.716; *p* = 0.003).

### 3.4 Post-baseline hospitalization duration

The median post-baseline hospitalization duration was significantly longer in the nirmatrelvir/ritonavir group than in the non-antiviral group [11.0 (IQR: 6.0–19.0) days vs. 9.0 (IQR: 5.0–14.0) days; *p* = 0.019] (Table 2). To assess whether longer hospitalization reflected a survivor effect or delayed recovery, we performed two supplemental analyses. First, a competing risks analysis (Supplementary Figure S1) showed a significantly lower cumulative incidence of death in the nirmatrelvir/ritonavir group (Gray’s test, *p* = 0.010), whereas the cumulative incidence of discharge did not differ between the groups (Gray’s test, *p* = 0.680), suggesting that the treatment reduced mortality without slowing discharge among survivors. Second, in severity-stratified analyses, the longer hospitalization duration was observed only in patients with critical illness [14.5 (IQR: 6.0–29.8) days vs. 9.0 (IQR: 4.0–15.0) days; *p* = 0.014] and not in those with severe illness [10.0 (IQR: 6.0–16.0) days vs. 9.0 (IQR: 6.0–13.0) days; *p* = 0.276; Supplementary Table S4]. Taken together, these findings suggest that the longer length of stay is most likely related to the mortality benefit of nirmatrelvir/ritonavir, as it reduced early deaths without delaying recovery among survivors.

### 3.5 Subgroup analyses

**Figure 3:**
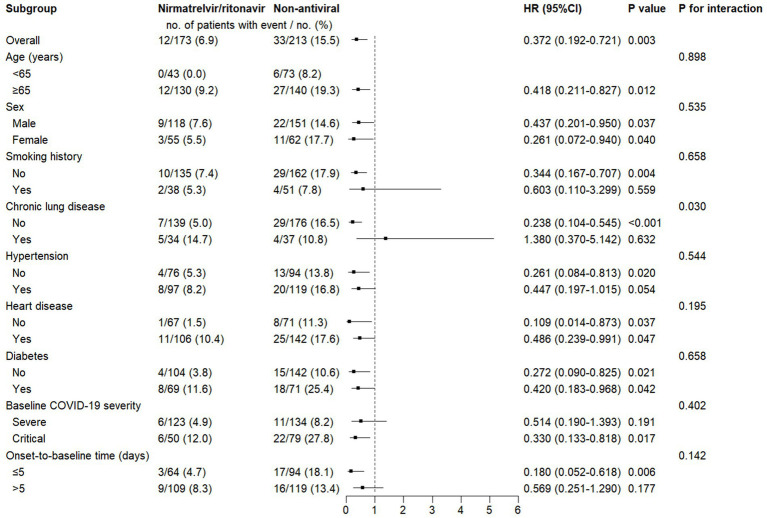
Subgroup analysis of the associations between nirmatrelvir/ritonavir and 28-day mortality. Categorical variables: Age (<65 or ≥65 years), sex (male or female), chronic lung disease (yes or no), hypertension (yes or no), heart disease (yes or no), diabetes (yes or no), baseline COVID-19 severity (severe or critical), and onset-to-baseline time (≤5 or >5 days). HR, hazard ratio; CI, confidence interval; COVID-19, coronavirus disease 2019.

Subgroup analyses for 28-day mortality are shown in Figure 3. Overall, the beneficial association of nirmatrelvir/ritonavir with mortality was consistent across most predefined subgroups. Notably, the point estimates suggested a substantial reduction in mortality risk among patients aged ≥65 years (HR = 0.418, 95% CI: 0.211–0.827), non-smokers (HR = 0.344, 95% CI: 0.167–0.707), those without chronic lung disease (HR = 0.238, 95% CI: 0.104–0.545), those without hypertension (HR = 0.261, 95% CI: 0.084–0.813), those with critical COVID-19 at baseline (HR = 0.330, 95% CI: 0.133–0.818), and those with an onset-to-baseline time of ≤5 days (HR = 0.180, 95% CI: 0.052–0.618). A significant interaction was observed between the treatment effect and chronic lung disease status (*P* for interaction < 0.05), suggesting that the treatment effect may differ according to this factor, with the benefit appearing more pronounced in patients without chronic lung disease. No significant interactions were identified for the other subgroup factors.

**Figure 4:**
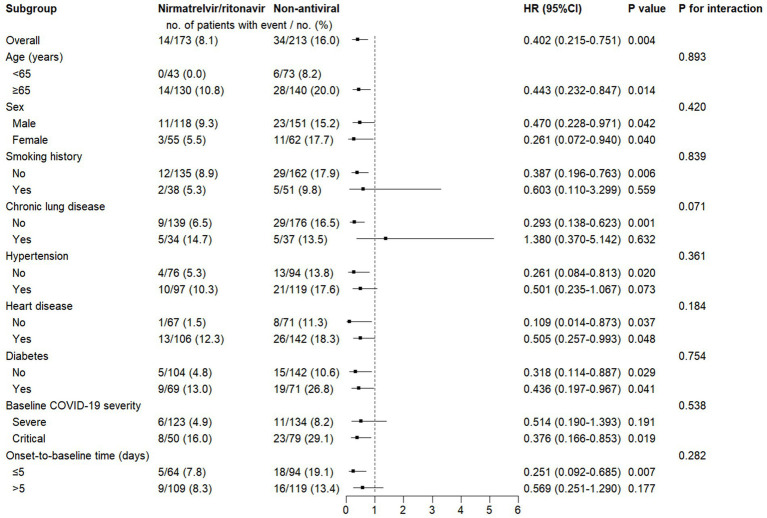
Subgroup analysis of the associations between nirmatrelvir/ritonavir and in-hospital mortality. Categorical variables: age (<65 or ≥65 years), sex (male or female), chronic lung disease (yes or no), hypertension (yes or no), heart disease (yes or no), diabetes (yes or no), baseline COVID-19 severity (severe or critical), and onset-to-baseline time (≤5 or >5 days). HR, hazard ratio; CI, confidence interval; COVID-19, coronavirus disease 2019.

Similarly, for in-hospital mortality (Figure 4), nirmatrelvir/ritonavir was associated with a reduced risk in the same subgroups. However, for this endpoint, no significant treatment-by-subgroup interactions were detected.

Taken together, these subgroup findings suggest that the survival benefit of nirmatrelvir/ritonavir was particularly evident in older patients (≥65 years) and in those without certain comorbidities, particularly chronic lung disease. In addition, the association was maintained in critically ill patients, and earlier initiation of treatment (within 5 days of symptom onset) was associated with a more pronounced benefit.

## 4 Discussion

This multicenter retrospective cohort study showed that treatment with nirmatrelvir/ritonavir was associated with a significant reduction in both 28-day mortality and in-hospital mortality among patients hospitalized with severe or critical COVID-19, and this association remained after adjustment for major potential confounders. At the same time, nirmatrelvir/ritonavir treatment was also associated with a longer post-baseline hospitalization duration.

Current evidence on the effectiveness of nirmatrelvir/ritonavir in hospitalized patients with severe or critical COVID-19 remains inconsistent. Studies in selected populations, such as mechanically ventilated patients or very old patients, have reported significant survival benefits (23, 24). In contrast, studies that included broader cohorts of severe or critical patients have not consistently shown a significant reduction in mortality (25, 26). These differences are likely related to important variations in study design and patient selection. Studies reporting positive outcomes were often based on relatively small samples or highly selected groups (23, 24). Conversely, studies reporting no benefit often did not exclude patients who had received other antiviral agents before baseline assessment or during follow-up (25, 26). Such pre-baseline or concomitant antiviral exposure could confound outcome assessment, potentially diluting the observed effect of nirmatrelvir/ritonavir and introducing bias.

Our study was designed to address these limitations and to provide less confounded evidence in this setting. First, we applied stringent exclusion criteria, excluding 205 patients with prior antiviral use and 194 who received other antivirals (including azvudine or molnupiravir) after the baseline date, which strengthened internal validity and allowed a clearer assessment of the treatment effect. Second, our cohort included a higher proportion of critically ill patients at baseline (33.4%) than that reported by Shao et al. (10.7%) (25), while Wang et al. (26) did not report this information. As disease severity is a well-established prognostic factor, the larger critically ill subgroup in our cohort may better reflect the real-world inpatient population for whom antiviral therapy is most clinically relevant and in whom a mortality signal may be more detectable. Therefore, our results, which showed a significant reduction in both 28-day mortality and in-hospital mortality, provide supportive evidence for the effectiveness of nirmatrelvir/ritonavir in severe and critical disease. Importantly, our findings align with earlier studies in selected high-risk populations while also offering a plausible methodological explanation for the negative results observed in some broader cohorts. Overall, these comparisons highlight the importance of careful patient selection and controlling concomitant antiviral exposure when evaluating antiviral efficacy in complex hospitalized settings.

The longer post-baseline hospitalization duration observed in the nirmatrelvir/ritonavir group also warrants careful interpretation. Our competing risks and severity-stratified analyses indicated that this finding does not reflect delayed recovery but is more likely related to the survival benefit of treatment. By effectively preventing early mortality, nirmatrelvir/ritonavir may increase the proportion of high-risk patients, particularly those with critical illness, who survive long enough to require a longer inpatient recovery period. From this perspective, a longer length of stay may reflect improved survival rather than a negative treatment effect.

Several limitations of this study should be considered. First, although antiviral agents act primarily by inhibiting viral replication, we did not perform serial daily viral load monitoring during hospitalization and, therefore, could not directly assess the effect of treatment on viral clearance. Second, vaccination status was missing from the clinical records of the majority of patients, which limited adjusting for its potential influence. Third, although this was a multicenter study, all hospitals were located within a single province, and the generalizability of our findings to other regions or healthcare systems with different patient profiles and standards of care may be limited. Despite these limitations, the eligibility criteria and the careful control of antiviral exposure strengthen the internal validity and support the reliability of the observed associations.

In conclusion, nirmatrelvir/ritonavir was associated with significantly reduced mortality in patients hospitalized with severe or critical COVID-19. These results support the use of nirmatrelvir/ritonavir as a therapeutic intervention in this high-risk population. However, to validate these findings and overcome the inherent limitations of retrospective studies, large multicenter randomized controlled trials focusing on severe and critical COVID-19 are still needed in order to provide higher-level evidence for guiding clinical decision-making and improving outcomes in this patient group.

## Data Availability

The raw data supporting the conclusions of this article will be made available by the authors, without undue reservation.
