# Supplementary material for: Nirmatrelvir/ritonavir reduced mortality in severe or critical COVID-19 patients: a multicenter retrospective cohort study
Source: Front Med (Lausanne). 2026 Feb 20;13:1747565. doi: 10.3389/fmed.2026.1747565 (PMC12962894; doi:10.3389/fmed.2026.1747565)
Supplement: Supplementary file 1 [file Supplementary_file_1.docx]

Supplementary Material

**Table S1** Definitions of COVID-19 severity categories according to the Diagnostic and Treatment Protocol for Novel Coronavirus Infection (Trial Version 10) issued by the National Health Commission of China

**Table S2** Univariate Cox regression analysis of 28-day mortality in patients with severe or critical COVID-19

**Table S3** Univariate Cox regression analysis of in-hospital mortality in patients with severe or critical COVID-19

**Table S4** Post-baseline hospitalization duration stratified by baseline COVID-19 severity

**Figure S1** Cumulative incidence functions for the competing events of discharge and death after baseline.

**Table S1** Definitions of COVID-19 severity categories according to the Diagnostic and Treatment Protocol for Novel Coronavirus Infection (Trial Version 10) issued by the National Health Commission of China

| COVID-19 severity | Definition |
| --- | --- |
| Mild | Patients primarily present with upper respiratory tract infection symptoms, such as dry throat, sore throat, cough, and fever. |
| Moderate | Persistent high fever (> 3 days) and/or cough, shortness of breath, etc., but with respiratory rate < 30 breaths/min and oxygen saturation > 93% on room air at rest. Characteristic imaging manifestations of COVID-19 pneumonia may be present. |
| Severe | Adults meeting any of the following criteria: |
|  | (i) Presence of shortness of breath with respiratory rate ≥ 30 breaths/min; |
|  | (ii) Oxygen saturation ≤ 93% on room air at rest; |
|  | (iii) Arterial partial pressure of oxygen (PaO₂) / oxygen concentration (FiO₂) ≤ 300 mmHg (1 mmHg = 0.133 kPa); |
|  | (iv) Clinical symptoms progressively worsening, with pulmonary imaging showing significant lesion progression (> 50%) within 24–48 hours. |
| Critical | Patients meeting any of the following criteria: |
|  | (i) Respiratory failure requiring mechanical ventilation; |
|  | (ii) Presence of shock; |
|  | (iii) Combined with other organ failure requiring ICU monitoring and treatment. |

Abbreviations: COVID-19, coronavirus disease 2019; ICU, intensive care unit.

**Table S2** Univariate Cox regression analysis of 28-day mortality in patients with severe or critical COVID-19

| Variables | HR (95%CI) | *P* value |
| --- | --- | --- |
| Nirmatrelvir/ritonavir | 0.372 (0.192–0.721) | 0.003 |
| Sex/female | 1.116 (0.593–2.099) | 0.734 |
| Age | 1.043 (1.019–1.067) | <0.001 |
| Smoking history | 0.532 (0.225–1.257) | 0.150 |
| Prior SARS-CoV-2 infection | 0.421 (0.058–3.067) | 0.393 |
| Chronic lung disease | 1.181 (0.568–2.452) | 0.656 |
| Hypertension | 1.085 (0.593–1.985) | 0.791 |
| Heart disease | 2.110 (1.016–4.381) | 0.045 |
| Cerebrovascular disease | 1.465 (0.807–2.659) | 0.209 |
| Diabetes | 2.083 (1.151–3.768) | 0.015 |
| Chronic liver disease | 0.579 (0.207–1.618) | 0.298 |
| Chronic kidney disease | 1.367 (0.691–2.701) | 0.369 |
| Cancer | 0.726 (0.337–1.562) | 0.412 |
| Immunocompromised status | 1.476 (0.457–4.769) | 0.515 |
| Critical COVID-19 at baseline | 3.039 (1.658–5.572) | <0.001 |
| Onset-to-baseline time | 1.004 (0.964–1.046) | 0.847 |
| Systemic glucocorticoids | 0.904 (0.457–1.786) | 0.770 |
| Antibiotics | 0.938 (0.392–2.243) | 0.886 |
| Tocilizumab | 3.491 (1.246–9.783) | 0.017 |
| Baricitinib | 1.277 (0.594–2.744) | 0.532 |
| Anticoagulant | 0.753 (0.388–1.461) | 0.402 |

Abbreviations: HR, hazard ratio; CI, confidence interval; SARS-CoV-2, severe acute respiratory syndrome coronavirus 2; COVID-19, coronavirus disease 2019.

**Table S3** Univariate Cox regression analysis of in-hospital mortality in patients with severe or critical COVID-19

| Variables | HR (95%CI) | *P* value |
| --- | --- | --- |
| Nirmatrelvir/ritonavir | 0.402 (0.215–0.751) | 0.004 |
| Sex/female | 1.016 (0.545–1.894) | 0.961 |
| Age | 1.045 (1.022–1.070) | <0.001 |
| Smoking history | 0.585 (0.262–1.305) | 0.190 |
| Prior SARS-CoV-2 infection | 0.651 (0.154–2.751) | 0.560 |
| Chronic lung disease | 1.281 (0.637–2.579) | 0.487 |
| Hypertension | 1.210 (0.669–2.189) | 0.529 |
| Heart disease | 2.197 (1.063–4.543) | 0.034 |
| Cerebrovascular disease | 1.471 (0.825–2.624) | 0.191 |
| Diabetes | 2.123 (1.194–3.773) | 0.010 |
| Chronic liver disease | 0.850 (0.361–2.001) | 0.710 |
| Chronic kidney disease | 1.597 (0.841–3.030) | 0.152 |
| Cancer | 0.618 (0.287–1.330) | 0.218 |
| Immunocompromised status | 1.476 (0.457–4.769) | 0.515 |
| Critical COVID-19 at baseline | 3.171 (1.743–5.770) | <0.001 |
| Onset-to-baseline time | 0.999 (0.960–1.040) | 0.978 |
| Systemic glucocorticoids | 0.853 (0.443–1.644) | 0.635 |
| Antibiotics | 0.945 (0.396–2.258) | 0.899 |
| Tocilizumab | 3.384 (1.209–9.471) | 0.020 |
| Baricitinib | 1.176 (0.550–2.516) | 0.675 |
| Anticoagulant | 0.726 (0.383–1.376) | 0.327 |

Abbreviations: HR, hazard ratio; CI, confidence interval; SARS-CoV-2, severe acute respiratory syndrome coronavirus 2; COVID-19, coronavirus disease 2019.

**Table S4** Post-baseline hospitalization duration stratified by baseline COVID-19 severity

| Severity at baseline | Group (n) | Post-baseline hospitalization  duration (days), M (IQR) | *P* value |
| --- | --- | --- | --- |
| Severe COVID-19 | Nirmatrelvir/ritonavir (n=123) | 10.0 (6.0-16.0) | 0.276 |
|  | Non-antiviral (n=134) | 9.0 (6.0-13.0) |  |
| Critical COVID-19 | Nirmatrelvir/ritonavir (n=50) | 14.5 (6.0-29.8) | 0.014 |
|  | Non-antiviral (n=79) | 9.0 (4.0-15.0) |  |

Abbreviations: M, median; IQR, interquartile range; COVID-19, coronavirus disease 2019.


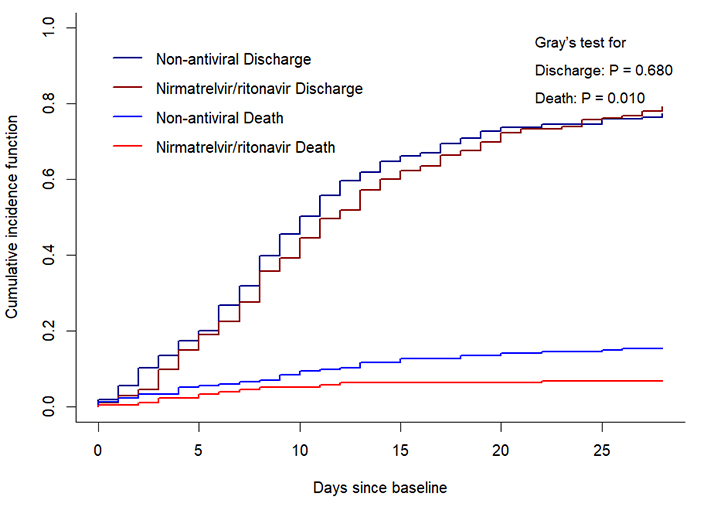


**Figure S1** Cumulative incidence functions for the competing events of discharge and death after baseline
